# Oral status and aesthetics after nonsurgical periodontal treatment: Do patient's perception and dentist's evaluation agree?

**DOI:** 10.1002/cre2.225

**Published:** 2019-08-19

**Authors:** Manuela Elena Kaufmann, Deborah Hofer, Daniel B. Wiedemeier, Thomas Attin, Patrick R. Schmidlin

**Affiliations:** ^1^ Clinic of Conservative and Preventive Dentistry, Center of Dental Medicine University of Zurich Zurich Switzerland; ^2^ Statistical Services, Center of Dental Medicine University of Zurich Zurich Switzerland

**Keywords:** aesthetics, objective, patient, perception, subjective, survey

## Abstract

**Objectives:**

Periodontal healing is often accompanied by side effects, which may cause an aesthetic deficit. The present investigation was focussed to compare patient's subjective perception of their posttherapy aesthetics with the objective measures of the results.

**Materials and methods:**

Survey results from patients (subjective parameters) on oral status and aesthetics were compared against routine clinical parameters and corresponding survey results from treating dentists (objective parameters), both before and after periodontal treatment. Subjective outcome parameters were then suitably transformed and compared with the objective ones to investigate the agreement between patients' perception and actual outcomes.

**Results:**

Objective recordings of periodontal status by the dentist and subjective awareness of the patient are quite contradictory to each other for almost all participants. Further, it was found that their aesthetics in the front were better after treatment, but dentist professionals targeted for future treatment needs.

**Conclusions:**

In this study, it was found that patients improved aesthetically on the upper jaw front after the therapy, which was not shared by the dentists. This discrepancy was due to the clinicians' view on more aesthetic corrective procedures than on patients' need.

## 1 INTRODUCTION

### 1.1 Background rationale

The primary goal for the prevention and therapy of periodontitis is the establishment and the preservation of the secondary oral health (Ramfjord, 1993). Reduction of inflammation and probing pocket depths, as well as gain of clinical attachment, are the main primary outcomes. These baseline clinical evaluations and subsequent re‐evaluations of the periodontal status are solely based on physical measurements of probing depth and/or attachment loss. These evaluations further underrecognize the true impact that periodontitis may have on the well‐being of the population (Papapanou & Susin, 2017). However, tissue shrinkage during the healing process is inevitable in most cases and often leads to a reduction of patient's quality of life (QoL), especially tissue shrinkage leading to recessions, which is an unavoidable side effect of the healing process in most cases and further may lead to a reduction of patient's perception of oral health‐related QoL (Mendez, Melchiors Angst, Stadler, Oppermann, & Gomes, 2017). Ferreira, Dias‐Pereira, Branco‐De‐Almeida, Martins, and Paiva (2017) found that periodontal disease, albeit less than gingivitis, can negatively impact oral health‐related QoL. When dental function and aesthetics become compromised, the clinician's evaluation of treatment needed and optimal therapy results may diverge from the patient's perception of these needs and the desired outcome of therapy. Despite actively informing patients of possible negative aesthetic side effects, including the risk of developing dentine hypersensitivity, patients may only realize what this means for them personally once treatment is completed, and respective additional therapy need may emerge (Schmidlin, 2012). Based on these risks, potential side effects, and the subjective clinical experience, the gut feeling of many dentists and hygienists remains that patients become overall healthier, but that unwanted and negative secondary side effects due to dentin exposure also inevitably lead to subjectively and objectively perceived aesthetic and functional impairments.

### 1.2 Objectives

The present study aimed to assess the subjective level of patient satisfaction after periodontal therapy using a questionnaire. Also, an objective survey for the dentist was provided to evaluate several clinical outcome measures at baseline and after nonsurgical periodontal therapy. Consequently, these data were compared to measure the level of agreement. The hypothesis considered in this study was that the objective results would not necessarily corroborate the subjective perception of the patient.

## 2 STUDY POPULATION AND METHODOLOGY

The Zurich Cantonal Ethics Committee evaluated the study, and a declaration of no objection was provided (BASEC Request No. 2017‐00984). Participation in the study was voluntary. Responses were considered as anonymous and used for research purposes only. Patients received oral as well as the written information regarding the research along with the methodology (including instructions for questionnaire). The age of the patients was not recorded because the survey was anonymous (Appendix S1), but the gender information was recorded. Table 1 presents the characteristics of the patient cohort. Observational cohort study was reported according to the STROBE guidelines (Von Elm et al., 2014).

**Table 1:** Characteristics of the patient cohort

Demographic data	n
Total	25
Gender	
Male	19
Female	6
Number of teeth (mean)	
Before treatment	24.28
After treatment	23.92
Smoking	
Nonsmoker	12
<10 cigarettes per day	9
>10 cigarettes per day	4
Carious lesions (mean)	
Before treatment	2.08
After treatment	0.64
Professional cleaning	
Never	10
>1 year but <2 years	4
>2 years	8
<1 year	3
Antibiotics from the dentist (with periodontal treatment)	
No	22
Yes	3

### 2.1 Study design

The questionnaire was designed on the basis of the guidelines provided by Williams (2003). Prior to the evaluation, the questionnaire was validated, and the investigators were recalibrated. Two questionnaires were designed for both patients as well as dentists separately. The first questionnaire was a subjective one for patients based on visual analogue scale (VAS), and the other one was an objective type for dentists on routine parameters as well as the indices related to periodontal screening (PSI) (Meyle & Jepsen, 2000), mobility (Isidor, 1998; Miller, 1950), phonetic aspects, percussion, sensitivity (Schiff et al., 1994), gingival recession (Miller, 1985), tooth color, halitosis, papilla level (Jemt, 1997), tooth gaps, abrasion (Parma, 1960), erosion (Lussi, 1996), systemic disease, and the number of remaining teeth. The following parameters were organized in four sections: concerning health, function, pain, and aesthetics (Tables 2 and 3).

**Table 2:** Subjective parameters in the patients' questionnaire

Subjective parameter	
Health	Health in general
	Tooth gap
Function	Tooth mobility and stability
	Speaking
	Chewing and biting
Pain	Sensitivity (air)
	Sensitivity (touching)
Aesthetics	Tooth color
	Halitosis
	Frontal upper jaw
	Teeth in general

**Table 3:** Objective routine parameters in the clinicians' questionnaire

Objective routine parameter		
Health	Systemic disease	From medical anamnesis
	Number of teeth	From oral status
	Maximum PSI (Williams, 2003)	PSI, periodontitis screening Per sextant → code number Per tooth, 6 values; the highest value code number gives the sextant overall code number 0healthy conditions 1BOP+ 2stain, uncontoured edges of restoration 3PD > 3–5 mm 4PD > 5 mm Corresponding USA: PSR (AAP, ADA 1992) Germany: PSI (DGP 2001) Switzerland: PGU (SSO 1999)
Function	Tooth mobility (Isidor, 1998; Meyle & Jepsen, 2000)	0 physiological 1 palpable horizontal 2 visible horizontal 3 elevated horizontal, additionally vertical
	Phonetic	wet pronunciation pronunciation errors such as lisping
	Percussion	Painful? (+/−)
Pain	Sensitivity (Miller, 1950)	Air Schiff score 0 no sensitivity ore sensation 1 barely perceptible sensitivity 2 mild pain 3 very discomforting pain
	Sensitivity (Schiff et al., 1994)	Gingival recession marginal tissue recession that does not extend to the mucogingival junction marginal tissue recession that extends to or beyond the mucogingival junction, with no periodontal attachment loss (bone or soft tissue) in the interdental area marginal tissue recession that extends to or beyond the mucogingival junction, with periodontal attachment loss in the interdental area or malpositioning of teeth marginal tissue recession that extends to or beyond the mucogingival junction, with severe bone or soft‐tissue loss in the interdental area and/or severe malpositioning of teeth
Aesthetics	Tooth color	VITA classical A1‐D4® shade guide
	Halitosis	Organoleptic measure no halitosis mildly, perceptible at a distance of 10 cm medium strong, perceptible at a distance of 30 cm strong, perceptible at a distance of 1 m
	Papilla (Miller, 1985)	hyperplastic papillae papilla fills up the entire proximal space half or more of the height of the papilla is present less than half of the height of the papilla is present no papilla is present
	Abrasion (Jemt, 1997)	0 no abrasion 1 loss of surface enamel 2 exposed dentine 3 involvement of secondary dentine 4 pulp exposure
	Erosion (Parma, 1960)	0 no erosion, enamel silky glazed appearance, absence of developmental ridges 1 loss of surface enamel, rounded cusps, edges of restorations rising above 2 involvement of dentine for less than half of tooth surface 3 involvement of dentine for more than half of tooth surface, erosion extending well into dentine and close to the pulp

Abbreviations: PGU, Parodontale Grunduntersuchung; PSI, Parodontaler Screening Index; PSR, Periodontal Screening Record.

### 2.2 Setting

The survey was conducted after periodontal examination and diagnosis and before the actual periodontal therapy started and was repeated after periodontal treatment, that is, 3 to 6 months after nonsurgical periodontal therapy. The study was pursued in the clinic of conservative and preventive dentistry, in the center of dental medicine, at the University of Zurich, in Zurich, Switzerland. All data were collected between October 13, 2017, and September 28, 2018. Patients were asked to rate the parameters on a horizontal (100 mm) VAS. The VAS was labelled for patients with the words “negative” on the left and “positive” on the right. The markings made by the patients were later measured with a ruler and given a rating of 0–10, based on the distance between the line's ends. The primary outcome measure of the subjective questionnaire was the mean change in VAS. As VAS score changes may seriously overestimate or underestimate changes resulting from treatment, a ±10‐mm tolerance was determined to equal a score of “no change.” All patients had a maximum time frame of 5 min to complete the questionnaire. The patient questionnaires were available in German, English, and Italian. The survey was constructed using simple, understandable sentences. Further, the well‐trained dentists were calibrated and involved in the student course. Two dentists have cross‐checked the data, and in case of any reported discrepancy, this was solved by discussion.

### 2.3 Participants

More than half of the patients lacked any academic background as well as had a low income. The surveys were returned anonymously. Each survey was numbered to match with the appropriate posttherapy study and the pretherapy and posttherapy clinician survey. The study did not consider a patient, in case a patient did not answer a question by keeping blank.

### 2.4 Variables

The questionnaires are shown in the Appendix S1 with all variables registered.

### 2.5 Bias

This pilot study was designed as an explorative survey and thus not rigorously controlled for sources of bias.

### 2.6 Study size

The final sample size for the pilot study was 25 patients who thoroughly answered the questions during the survey. The first 25 patients that completely answered the survey questions were included.

### 2.7 Statistical methods

In the present investigation, descriptive statistics as well as the graphical presentation were used to illustrate the changes between before and after periodontal treatment. The subjective perceptions from the patients were assessed through VAS and were categorized into “improved,” “no change,” and “worsened.” Similarly, the objective parameters, evaluated on a categorical scale, lead naturally to these outcome categories. Due to the continuous nature of the VAS, a ±10‐mm tolerance was determined to equal a score of “no change.” All analyses and graphics were computed with the statistical software R (Team, 2018), including the package ggplot2 (Wickham, 2016).

## 3 RESULTS

### 3.1 Participants

Fifty‐two patients have participated in the survey, and 25 patients (19 men and 6 women) were included in this study based on the criteria mentioned in the previous section.

### 3.2 Descriptive data

**Figure 1:**
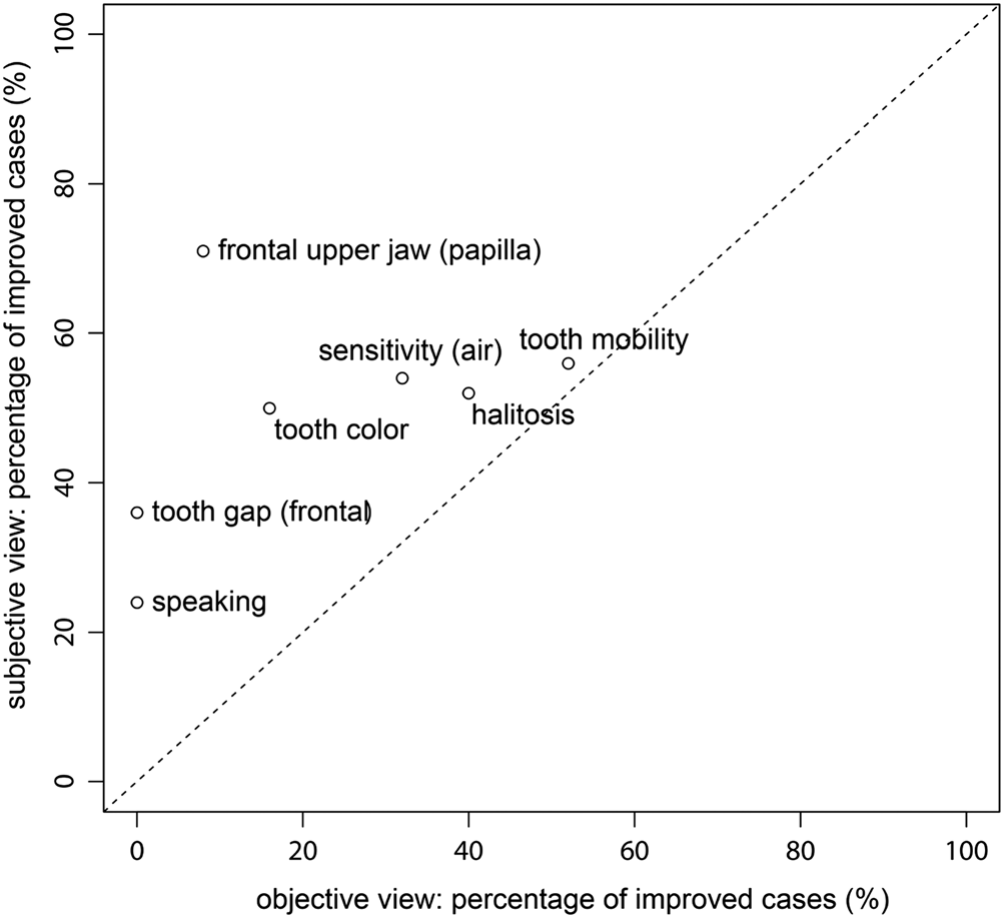
Percentage of subjective and objective views of improved cases

Figure 1 envisages the percentage of improved cases subjectively and objectively and illustrates clearly that the patient's perception and clinician's evaluation disagree. Tooth mobility is the only parameter that most correlated among all.

### 3.3 Outcome data

**Figure 2:**
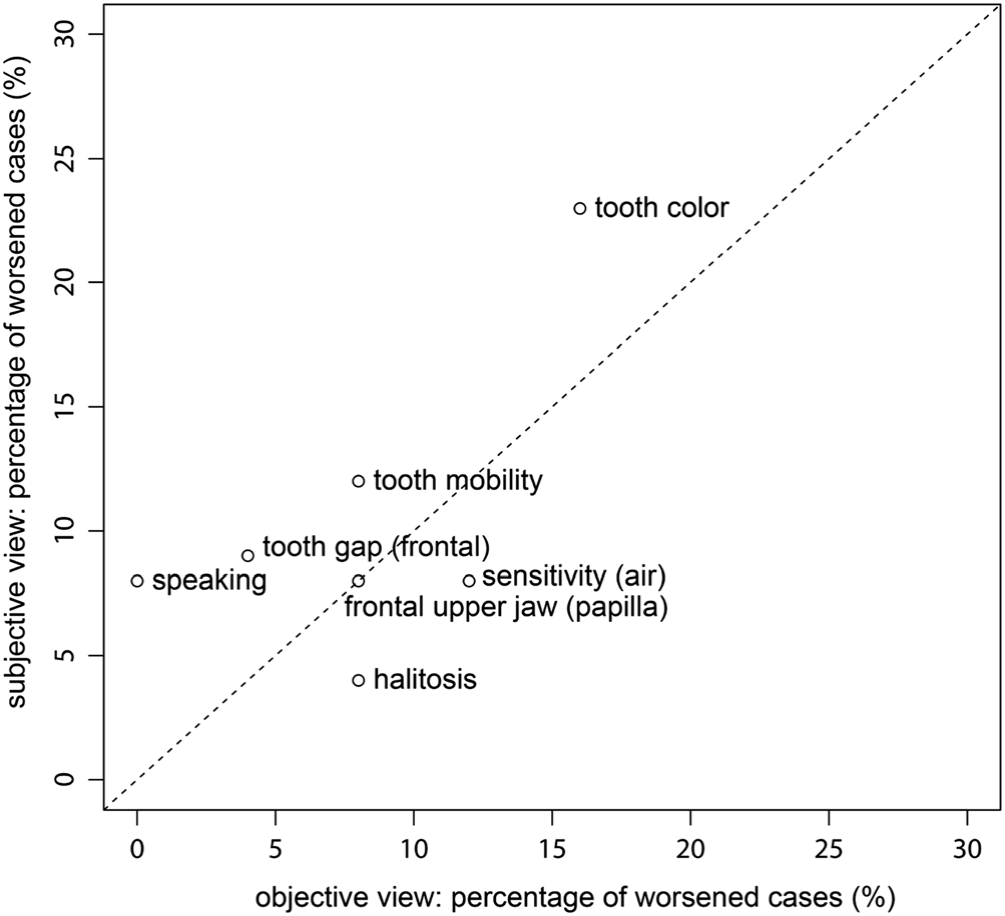
Percentage of subjective and objective views of worsened cases

In general, the results and percentage of improved cases are located more in the field of the subjective view, which indicates that patients perceived well compared with the objective‐based evaluation by dentists. Further, results showed the least correlated cases between them were slightly higher (Figure 2).

### 3.4 Main results

In the following sections, we briefly report on the characteristics of the individual patient along with the results based on the single evaluation criteria.

#### 3.4.1 General and oral health

Thirteen patients were initially diagnosed with systemic diseases. Before treatment, patients displayed a median number of teeth of 25 (first quartile, 24; third quartile, 27). After therapy, six patients had to extract teeth due to a hopeless prognosis (median, 25 teeth; first quartile, 21; third quartile, 27). All patients had a minimum PSI (Meyle & Jepsen, 2000) of 3 (1) or 4 (24). After therapy, the PSI improved (9) or was stable (16).

#### 3.4.2 Oral function

Tooth mobility increased subjectively as well as objectively, which was also reported earlier in the literature (Isidor, 1998; Miller, 1950). Only for two patients did tooth mobility objectively decrease.

However, the objective results for the evaluation of the phonetic status before and after therapy did not alter, but 24% of the patients found to have improved ability to speak. The masticatory function of patients with extracted teeth was reduced after therapy due to minimum chewing units or antagonist pairs, whereas all other patients reported a better impression of their chewing function.

#### 3.4.3 Pain

The objective evaluation of percussion remained unchanged in 21 patients but responded affirmatively to the percussion test after therapy of two patients on one tooth each. Dentin hypersensitivity was tested and improved in the majority of the cases, and one fifth of the patients reported suffering from increased sensitivity after therapy. Similarly, objectively, hypersensitivity due to gingival recession after treatment was found for only one patient, and a similar study was reported in the earlier literature (Miller, 1985). Further, tooth sensitivity, tested by the application of compressed air, was reduced in three cases but remained stable in 14 cases (Schiff et al., 1994).

#### 3.4.4 Aesthetics

**Figure 3:**
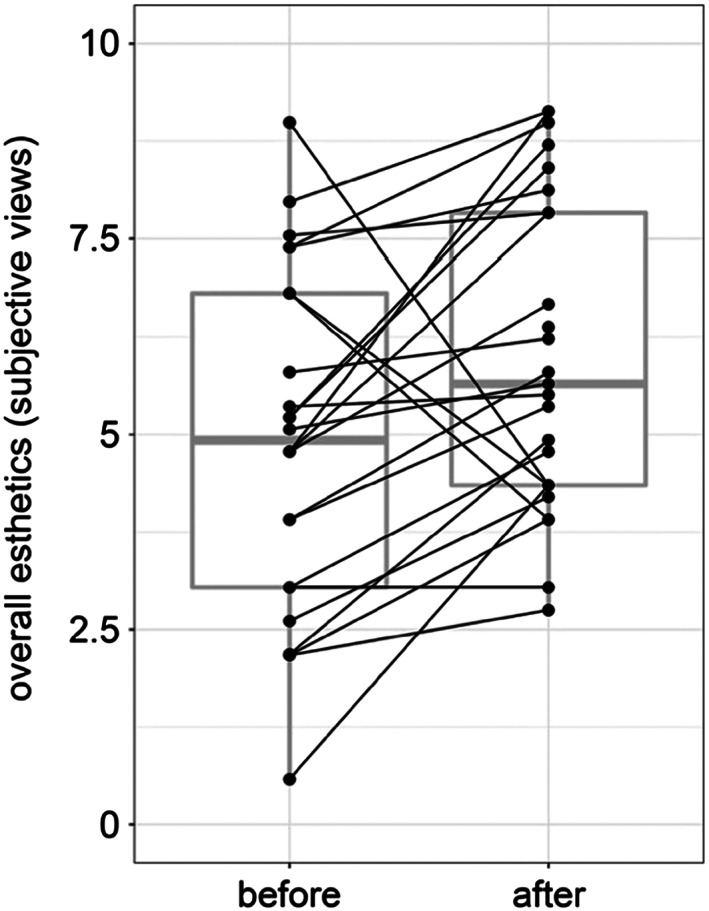
Subjective views of the patients as ticked on the visual analogue scale before and after nonsurgical periodontal treatment

**Figure 4:**
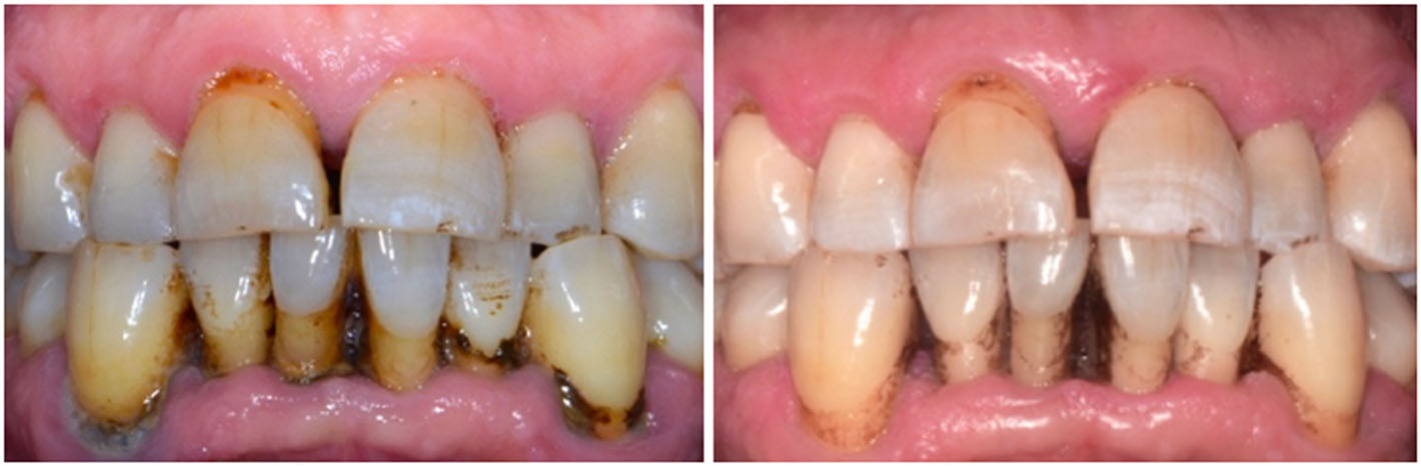
After therapy (right), the patient recognized neither gingival shrinkage nor gingival recession. The “black holes” between teeth 41/31 did not bother the patient. Before treatment (left), the discoloration (plaque and stain) did not worry the patient. After nonsurgical periodontal therapy (right) and rinsing with 0.2% chlorhexidine mouth rinse, the patient registered the discoloration of the teeth

More than 85% of the patients were not concerned about tooth gaps before or after periodontal therapy, but all patients revealed gaps in the posterior dentition. Most patients were not bothered by the color of their teeth pretreatment. However, one fifth of the patients became disturbed after therapy. The simultaneous gingival shrinkage was not recognized by all patients and is depicted in Figure 4. Except for one participant, all patients subjectively evaluated themselves as having halitosis after periodontal therapy. The objective assessment for two patients, after treatment, however, also revealed a persisting lousy breath. Of the 23 patients who evaluated themselves as having oral malodor, 10 were objectively evaluated as being malodor free, whereas for the remaining 13 patients, the organoleptic halitosis level remained unchanged. The papillary situation (Jemt, 1997) in the frontal teeth remained stable in 21 patients. Seventeen patients subjectively found the aesthetics in the front to be better after treatment. Abrasion (Parma, 1960) remained unchanged in 20 patients. One patient was evaluated, objectively, to have grade 2 dentin exposure. The erosion grade (Lussi, 1996) remained constant for 16 patients, whereas for six patients, the value lowered and but increased trend for three patients. Boxplot presentation of subjective patient views of the overall aesthetics before and after therapy is shown in Figure 3.

## 4 DISCUSSION

### 4.1 Interpretation

The results of the present study confirm that the selection of the best procedure for a particular patient to treat in dentistry could be a daunting task for experienced practitioners. Further, the clinical outcomes and patient's perception, which is also a real outcome (Tsakos, Allen, Steele, & Locker, 2012), ideally match but deviates in a few cases. Periodontal treatment will most probably fail if one part fails (westfelt, 1996).

### 4.2 Generalizability

Based on the survey, report on patient may provide keener insight into an individual's perspectives and experiences, to assist in decision‐making process during the treatment. For most of the cases, it was a better option to evaluate a new patient on the basis of the knowledge of previous patient's experiences and perspectives, satisfaction, and oral health‐related QoL data. Furthermore, the survey research was reliable as the outcome was based on the practical data reported by the patient. Self‐reporting has been consistently shown to be more accurate than proxy reporting (Fowler, 1995). The validity of a study depends in a large part on the response rate, as a poor response rate can result in a nonresponse bias. The results of a self‐perception longitudinal study (Stadler, Romagna, Rossi, Costa, & Gomes, 2017) after periodontal treatment showed a favorable perception related to the treatment and continued satisfaction for over time. The patients had to fill in a questionnaire within a total 40 items related to the knowledge of changes in clinical signs of periodontal disease, psychological aspects on oral health status, and satisfaction with the treatment. The questionnaires though were not compared with objective findings as we did in our study. As questionnaire response, they used a Likert scale ranging from 1 to 5 points and dichotomized the results for each question into 1 or 0, respectively, showing if the subject was favorable or unfavorable to treatment. In our study, we used the VAS as response option. First published was the VAS in the early 1920s and is often used to measure pain (Hayes, 1921). Typically, the scale is used in horizontal format and enable the patient a more exceptional distinction between subjective states to be made (Aitken, 1969). However, it has also been found that patients find it difficult to judge how to rate (Carlsson, 1983). The reason for the high dropout rate in our VAS may be due to the minimum time frame to complete as well as the intellectual level of the patients. Also, there were no further standardized instructions sought to complete the questionnaire except to fill in as it was self‐explanatory.

Brauchle, Noack, and Reich (2013) found that periodontal disease has influenced the oral health‐related QoL. The German version of Oral Health Impact Profile was applied. In 2017, Mendez, Melchiors Angst, Stadler, Oppermann, and Gomes (2017) applied Oral Health Impact Profile 30 days before and 90 days after nonsurgical periodontal treatment and concluded a significant improvement in the oral health‐related QoL. Further, women noticed a higher positive impact on their social environment (*ρ* < .05) after systematic periodontal therapy than men. By this treatment, patient's complaints were reduced (*ρ* < .001) (Franke, Bröseler, & Tietmann, 2015). The patient satisfaction was generally defined as a perceived value judgment and sustained the response to service‐related stimuli before, during, and after use of the service (Aharony & Strasser, 1993).

VAS is analyzed as noncontinuous using statistical methods for ordinal data (Lund et al., 2005). When VAS is treated as an interval‐scaled data for sample size calculations in clinical trials, as a consequence, it will lead to inappropriate conclusion of trials. Thus, incorrect analyses, using parametric statistics on VAS data, may have implications for the interpretation of the effectiveness of interventions and services. The VAS was considered as a more straightforward way to ascertain the patient's perception of the aesthetics. Also, it was a quick method with high reproducibility (Heravi, Rashed, & Abachizadeh, 2011; Hirvinen, Heikinheimo, & Svedström‐Oristo, 2012; Ioi, Nakata, & Counts, 2010).

### 4.3 Limitations

Successful outcomes in dentistry, as also in plastic surgery, are often measured by improvement in a patient's QoL rather than by mortality rates, which are used by other medical areas. Improved general and oral health through nonsurgical periodontal treatment (Chapple et al., 2013) were considered in this study and explained by the fact that patients attended several appointments during the procedure. The frequency of visits could apart from objective parameters observed and also have provided them with feeling responses. The repeated motivation for performing oral health care at every appointment might have had an impact on leading a healthier lifestyle in general, but psychosocial conditions might alter the host immune response and thus predispose individuals to periodontal disease (Preeja, Ambili, Nisha, Seba, & Archana, 2013).

The rehabilitating of oral function through nonsurgical periodontal therapy included in some cases and also to extract teeth at the beginning of periodontal treatment was mainly due to many reasons. Fractures, advanced periodontal lesions, and severely increased mobility was found to be not worth preserving due to further reconstructive planing or required root canal revision for which the cost was not justified, even at the reduced rate incurred by dental students in their student clinic. For nonprofessionals (patients), a distinct oral functional parameter such as tooth mobility is easy to assess as clinicians do (Figure 1). A study in the United States of Eke and Dye (2009) found that self‐report oral health measure is a promising tool for prediction of the population prevalence of periodontitis. Similar observations underlined in the present study on the telephone interviews on self‐reporting questions regarding gum disease, loose teeth, and tooth appearance found the highest sensitivity in surveillance, but the screening of periodontitis was compared with the clinical findings.

Regarding pain, the placebo effect could play a role in respect of sensitivity (Schmidlin & Sahrmann, 2013). Objectively, sensitivity after scaling is a common finding, whereas lowered sensitivity may be explained in reducing the inflammatory load. The term “root sensitivity” was suggested by the European Federation of Periodontology (Von Troil, Needleman, & Sanz, 2002) to describe tooth sensitivity associated with periodontal disease and its treatment. Inconsistencies in the recording of sensitivity, diverse duration of studies, and type of therapy that was provided do not allow to conclude a reviewing incidence for sensitivity after periodontal therapy. In literature, the incidence ranges from 23% to 80.4%, peaking about 1 week following periodontal therapy (Lin & Gillam, 2012).

### 4.4 Key results

From Figure 4, it can be observed that a recession is aesthetically not always the first thing patients do care up to the invisible of recession during smiling. Because recession depth is measured with a periodontal probe positioned between the cemento enamel junction (CEJ) and the gingival margin, it is clear that the detection of the CEJ is key for this measurement. In some patients, the CEJ was covered by a cervical restoration, and recession diagnosis was possible but difficult. Patients were carefully and several times instructed for proper hygiene. Improper toothbrushing might influence the development and progression of gingival recession, which is inconclusive from data (Heasman, Holliday, Bryant, & Preshaw, 2015) and not for occlusal forces (Harrel & Nunn, 2004). The color as another aesthetic parameter can be improved through cleaning; on the other hand, teeth darkening/discoloration can be improved temporarily through rinsing with 0.2% chlorhexidine mouth rinse solution or betadine (antiseptic 10% solution povidone‐iodine) mouth rinse, which all patients got for 10 days to rinse with twice daily for 1 min in addition to daily oral care after scaling/root planing. Tooth color is another distinct parameter that patients judge immediately when it changes.

Further, the bleaching study showed does have an impact on the QoL as self‐confidence increased after bleaching after followed up after 2 years by the patient (Bersezio et al., 2019). Even though the papilla situation worsened, it was rated inadequate when it functionally disturbed patients because of food impaction or wet pronunciation. People sometimes also refer proximal space and find tooth color more critical. The “negative” alteration of three patients that worsened to erosion grade (from Grade 0 to Grade 1) must also be justified through the eyes of the treater. Also, to distinct erosion from abrasion may not be in every case very evident. The multifactor nature of tooth wear compounds on the loss of surface substance and the loss of a tooth was mainly reported for erosion, attrition, abrasion, and abfraction (Nunn, 1996). The diagnostic procedure of decay is a visual rather than instrumental approach. Also, the abrasion level of the tooth in this study is objectively improved due to extraction.

## 5 CONCLUSION

The clinicians are faced with a broad spectrum of therapeutic options during treatment. Therapy should avoid focus on objective parameters such as pain reduction and also the subjective perceivements of patients. The patients' subjective perceivements are crucial for a happy outcome of treatment for a patient point of view, but the cure is the wanted outcome of the clinician. Also, the objective parameters should be well defined before the treatment, along with the subjective parameters of the individual patient.

## CLINICAL RELEVANCE

### Scientific rationale for the study

The healing aspect after periodontal therapy may influence the patient's perception of the individual outcome. The main side effects against this may be the recession formation or the dentin hypersensitivity. The present study focussed on the assessment of the agreement between the results anticipated by the patient and the objective clinical parameters.

### Principal findings

The objective and subjective need for aesthetic or functional treatment did not seem to agree 100%. The parameter that is perceived as the most similar objectively by clinicians and patients alike is tooth mobility. The adverse outcomes are in general and less problematic for patients than one may believe.

## AUTHOR CONTRIBUTIONS

M. E. K. drafted the survey outlines and the manuscript. D. H. helped in developing the surveys and the manuscript. D. B. W. verified the analytical methods, helped with the statistical evaluation and the graphics, and participated in its design. T. A. validated the manuscript. P. R. S. conceived the study and supervised the study. All authors carefully read and approved the final text.

## FUNDING

This research received no external funding.

## CONFLICT OF INTERESTS

The authors declare no conflict of interests.

## Supporting information


**Data S1:** Supplementary Information
