# Supplementary material for: Oral status and aesthetics after nonsurgical periodontal treatment: Do patient's perception and dentist's evaluation agree?
Source: Clin Exp Dent Res. 2019 Aug 19;5(6):601–10. doi: 10.1002/cre2.225 (PMC9514215; doi:10.1002/cre2.225)
Supplement: Supplementary file 1 — Data S1: Supplementary Information [file CRE2-5-601-s001.docx]

**Zusatzfragebogen Patient**

- Lieber Teilnehmer – vielen Dank, dass Sie an dieser Studie teilnehmen!
- Im Rahmen unserer Studie zur subjektiven Wahrnehmung der Patienten vor und nach Parodontitis-Therapie, möchten wir Sie bitten diesen Fragebogen zu bearbeiten.
- Füllen Sie bitte den ganzen Fragebogen aus und lassen Sie keine Frage aus.
- Sie haben **5 min** Zeit.
- Bitte Ihre Antwort mit einem Stift auf der Linie exakt markieren. Beispiel:

1a) Wie zufrieden sind Sie mit der **Ästhetik** Ihrer Zähne?

extrem unzufrieden sehr zufrieden

Oberkiefer Front

Unterkiefer Front

Gesamt

1b) **Was stört** Sie ästhetisch am meisten?

extrem störend gar nicht störend

Zahnfarbe

Zahnform

Lücken

Anderes:..................

.................................

1. Haben Sie **Mundgeruch** und wie stark beurteilen Sie diesen?

extrem störend gar nicht vorhanden

Mundgeruch

1. Haben Sie **Schmerzen**?

massiv überhaupt nicht

Beim Essen/Kauen

Auf Luft

Beim Zähneputzen

Spontan

1. Wie beurteilen Sie die **Funktion** Ihrer Zähne?

geht gar nicht funktioniert bestens

Abbeissen

Kauen

Reden/Sprechen

Zahnbeweglichkeit/

Stabilität

1. Wie beurteilen Sie Ihre **Gesundheit**?

massiv beeinträchtigt absolut gesund

Gesamter

Organismus/Körper

Mund/Rachenraum

Zähne

1. Hatten Sie je zu einem früheren Zeitpunkt bereits eine **parodontale Behandlung**?

Wenn JA: Wann? (Jahr, Monat, Kanton, Land).............................................

Wo? Bei Dentalhygienikerin

Beim Allgemeinzahnarzt

Beim Spezialisten (Parodontologe)

Mit Antibiotika Ohne Antibiotika?

**Zusatzfragebogen Untersucher**

1a) **Ästhetik**

**Papillen** (Index nach JEMT 0-4)

4 hyperplastische Papillen

3 die Papille füllt den gesamten Approximalraum aus

2 die Hälfte oder mehr der Höhe der Papille ist erhalten

1 weniger als die Hälfte der Höhe der Papille ist erhalten

0 keine Papille

**Mittellinienabweichung OK/UK** (in mm) : .......................

**Lachlinie** (hoch-mittel-tief, je höher oder tiefer desto ungünstiger)

hoch mittel tief

**Zahnfarbe** (VITA classical) : .......................

**Zahnstellung**

Kippungen

Elongationen

Engstand

Lücken im Seitenzahngebiet

Lücken in der Front

1. **Mundgeruch** (organoleptische Messung)

0 kein

1 leicht, bei 10 cm Abstand perzeptierbar

2 mittelstark, bei 30 cm Abstand perzeptierbar

3 stark, bei 1 m Abstand perzeptierbar

1. **Schmerzen**

**Abrasionen** (nach PARMA 0-4)

0 keine Abrasion

1 im Schmelzbereich

2 Dentin liegt frei

3 sekundäres Dentin liegt frei

4 Eröffnung der Pulpa

**Erosionen** (nach LUSSI 0-3)

0 keine Erosion, kein Schmelzverlust, Schmelz glänzend, Verlust der Perikymatien möglich

1 beginnender Verlust von Oberflächenstruktur, Schmelz zeigt Seidenglanz, intakte Schmelzleiste zervikal, gerundete Höcker, Füllungen höher; Stufenbildungen, Eindellungen

2 deutliche Schädigung, Schmelzverlust <50%, freiliegendes Dentin

3 = 2, Schmelzverlust >50%

**Zahnempfindlichkeit** (mit Luftstoss, SCHIFF Score 0-3)

0 keine Empfindlichkeiten

1 leicht empfindlich

2 mittel empfindlich

3 sehr empfindlich

**Rezessionen** (nach MILLER 1-4)

1 nicht bis Mukogingivallinie, kein Gewebe- oder Knochenverlust

2 bis Mukogingivallinie, kein Gewebe- oder Knochenverlust

3 bis Mukogingivallinie, Gewebe- oder Knochenverlust, Zahnfehlstellung

4 bis Mukogingivallinie, Gewebe- oder Knochenverlust, schwere Zahnfehlstellung

1. **Funktion**

**Klopfdolenzen**: Perkussion positiv Perkussion negativ

**Phonetik** gestört: ja nein

**Zahnmobilität** (nach FLEMMING 0-3)

0 physiologisch

1 spürbar horizontal

2 sichtbar horizontal

3 erhöht horizontal, zusätzlich vertikal

1. **Allg. Gesundheit**

**Anzahl Zähne:**

**Anzahl Zähne mit Karies:**

**Max. PGU:**

**Mundschleimhaut-Veränderung:** ja nein

.......................

**Speichel:** genug mangelhaft

**Systemische Erkrankung:** ja nein

..................................

**Rauchen:** ja > 10/d ja < 10/d nein
